# HoloKinect: Holographic 3D Video Conferencing

**DOI:** 10.3390/s22218118

**Published:** 2022-10-23

**Authors:** Stephen Siemonsma, Tyler Bell

**Affiliations:** Department of Electrical and Computer Engineering, University of Iowa, Iowa City, IA 52242, USA

**Keywords:** 3D video conferencing, 3D video streaming, depth encoding, telepresence, Microsoft Kinect, multiscopic displays, virtual reality, augmented reality

## Abstract

Recent world events have caused a dramatic rise in the use of video conferencing solutions such as Zoom and FaceTime. Although 3D capture and display technologies are becoming common in consumer products (e.g., Apple iPhone TrueDepth sensors, Microsoft Kinect devices, and Meta Quest VR headsets), 3D telecommunication has not yet seen any appreciable adoption. Researchers have made great progress in developing advanced 3D telepresence systems, but often with burdensome hardware and network requirements. In this work, we present HoloKinect, an open-source, user-friendly, and GPU-accelerated platform for enabling live, two-way 3D video conferencing on commodity hardware and a standard broadband internet connection. A Microsoft Azure Kinect serves as the capture device and a Looking Glass Portrait multiscopically displays the final reconstructed 3D mesh for a hologram-like effect. HoloKinect packs color and depth information into a single video stream, leveraging multiwavelength depth (MWD) encoding to store depth maps in standard RGB video frames. The video stream is compressed with highly optimized and hardware-accelerated video codecs such as H.264. A search of the depth and video encoding parameter space was performed to analyze the quantitative and qualitative losses resulting from HoloKinect’s lossy compression scheme. Visual results were acceptable at all tested bitrates (3–30 Mbps), while the best results were achieved with higher video bitrates and full 4:4:4 chroma sampling. RMSE values of the recovered depth measurements were low across all settings permutations.

## 1. Introduction

For decades, researchers have been envisioning and developing 3D telepresence systems [1,2,3,4,5,6,7,8,9]. At first, 3D telepresence was mostly a thought experiment [1] that the technology of the time was unable to realize. Eventually, full systems leveraging new 3D display technologies were developed, although they could not yet run with high frame rates [2]. More recently, 3D capture technology and methods have matured significantly and modern computer hardware now allows 3D telepresence systems to run at reasonably high frame rates [8,9]. Although older systems focused on large 3D displays that required polarized or actively shuttering glasses [2], technology trends are now moving towards more self-contained 3D display technologies such as augmented reality (AR) and virtual reality (VR) headsets [7,10], glasses-free autostereoscopic displays [3,9,11], and even multiscopic displays [12].

Researchers and product developers now have access to a multitude of well-established 3D capture methods, including passive stereo, active stereo, structured light, and time-of-flight (ToF) techniques [13]. State-of-the-art implementations [8,9,14] are now focusing on the fusion of depth and texture information from multiple cameras with increasingly high audiovisual fidelity, with the goal of making the rendered 3D image look as natural and realistic as possible. These multi-sensor systems can also allow the spectator to be tracked in real time so that they may view the remote subject from a variety of different perspectives. Additional focus has also been placed on facilitating non-verbal forms of communication such as eye contact [3,5,9] and body language [9]. A recent state-of-the-art system, Project Starline [9], achieved a critical milestone by being the first 3D telepresence system that participants rated more favorably than standard 2D video conferencing.

Current state-of-the-art systems [7,8,9] yield impressive results, but the size, complexity, cost, and specialized nature of these types of installations currently prevent them from being practical in a typical office or home setting. Fortunately, many other 3D telepresence systems have been able to leverage off-the-shelf hardware such as Microsoft Kinect devices [5] and Apple’s iPhone TrueDepth sensor [15]. Additionally, some algorithms have focused on optimizing the compression of the data to allow for low-bandwidth 3D streaming [8,15]. Although it is possible to compress and stream 3D geometry directly [8], many bandwidth-efficient implementations [9,15] have compressed color and depth (RGB-D) data using 2D video compression schemes (e.g., H.264, H.265), which are heavily optimized to exploit spatial and temporal redundancies. Dedicated hardware video encoders and decoders are commonly present on modern devices, lessening the computational burden when using this method of compression.

The COVID-19 pandemic has brought about a rapid transition to remote work [16], education [17], and socialization. Conventional 2D video conferencing applications such as Zoom and FaceTime have been integral to this evolution in culture. That said, 3D telepresence systems have not yet found any widespread adoption, despite the dramatic rise in consumer-level hardware capable of 3D capture and display. This is partly due to the burdensome hardware, processing, and bandwidth requirements of the 3D telepresence systems that rely on complex, multi-sensor 3D capture systems. While these types of systems are instrumental for pushing the boundaries of photorealistic 3D telepresence, their requirements are prohibitively costly and complex for the general consumer.

In this work, we present HoloKinect. HoloKinect is an end-to-end 3D video conferencing solution that leverages off-the-shelf hardware and software technologies, all streaming over a conventional internet connection at a user-configurable target bitrate. Our system is built entirely in the Unity game engine, resulting in a highly portable, self-contained executable project with a multitude of visualization options. The current implementation uses a Microsoft Azure Kinect for RGB-D capture and a Looking Glass Portrait multiscopic display for 3D visualization at each end of the two-way video conference, enabling a hologram-like effect. The implementation is highly modular and easily facilitates alternative RGB-D capture systems (e.g., iPhone TrueDepth sensor) or 3D display technologies (e.g., VR/AR head-mounted displays). The system runs on Windows PCs for the best compatibility with the currently implemented 3D capture and display hardware, but the underlying compression, streaming, decompression, and postprocessing framework can be compiled to most of the device types supported by the Unity game engine.

To enable the efficient streaming of high-resolution RGB-D data, we use the multiwavelength depth (MWD) [18] method to encode each 16-bit depth map into a standard 2D color image. Each MWD encoding and its corresponding color texture are packed into a composite video frame, which is then H.264 video compressed and transmitted with WebRTC [19] to a remote client via the Agora video streaming platform [20]. At the remote client, the composite video frames are received and decompressed. The depth data is further decoded using MWD. The RGB-D data is then used to construct a mesh, which is further postprocessed with a GPU-accelerated pipeline to filter depth discontinuities and other visually distracting data, fill holes, smooth the depth map, and sharpen the color texture. Unity’s renderer then applies lighting effects, and finally the Looking Glass SDK [21] renders 48 distinct views, each at a resolution of 420 × 560, on the Looking Glass Portrait display at a steady 30 frames per second (FPS).

The remainder of this paper describes the HoloKinect 3D video conferencing system. Section 2 describes the system as a whole and details each of its main components: Acquisition, Preprocessing, Depth Encoding, Video Streaming, Depth Decoding, Postprocessing, and 3D Visualization. Section 3 shows qualitative examples and quantitative experimental results that explore the performance of HoloKinect across a parameter space of depth encoding and video compression settings. Section 4 provides additional experimental insight into the selection of various encoding parameters, in addition to a discussion on the strengths, limitations, applications, and future directions of this work. Section 5 will summarize and conclude the paper.

## 2. Materials and Methods

### 2.1. System Overview

**Figure 1:**
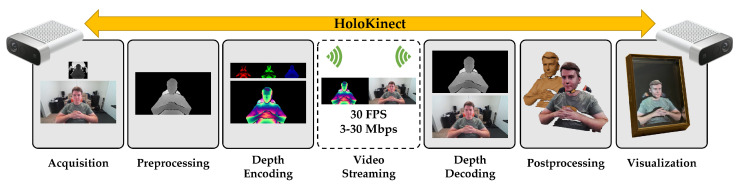
HoloKinect, an end-to-end solution for 3D video conferencing with a simulated holographic effect. The current implementation uses a Microsoft Azure Kinect for RGB-D capture and a Looking Glass Portrait multiscopic display for 3D visualization at each end of the live two-way video conference. The implementation is highly modular and easily facilitates alternative RGB-D capture systems (e.g., iPhone TrueDepth sensor) or 3D display technologies (e.g., VR/AR head-mounted displays).

An overview of the HoloKinect pipeline can be seen in Figure 1. This pipeline is currently designed to have only modest system requirements on the sending side, while the receiving side can employ a scalable and highly customizable set of postprocessing and visualization strategies. The first step is color, depth, and sound data acquisition, facilitated by a Microsoft Azure Kinect. The depth and color maps are preprocessed by transforming them into a shared perspective and resolution, with the depth map being thresholded to a desired depth of field. We employ multiwavelength depth (MWD) [18] to encode the 16-bit depth values into a standard RGB color image. This MWD encoding is then stitched side-by-side with the corresponding color texture image to form a composite frame. This frame is video compressed and transmitted alongside the audio using Agora, an off-the-shelf WebRTC solution. On the receiving end, the MWD encoding is decoded to recover the depth map. A multitude of GPU-accelerated postprocessing options are available at this point to improve the perceived visual quality. Finally, the depth and color maps are combined into a single 3D mesh using the Unity game engine, which applies perspective visualization, shadows, and lighting effects. Unity interfaces with a Looking Glass Portrait to render the final holographic 3D image. By simultaneously running on two remote devices, HoloKinect achieves seamless two-way 3D video conferencing over a standard internet connection with hologram-like visualizations.

### 2.2. Acquisition

The Microsoft Azure Kinect is equipped with a 1-megapixel amplitude-modulated continuous wave (AMCW) time-of-flight (ToF) depth camera. It uses a pair of near-infrared laser diodes, allowing it to illuminate the scene using either a narrow or wide field of view mode. An indirect measure of the time of flight of the IR light received at each pixel location is used to generate a depth map. The Azure Kinect supports a variety of color and depth sensor capture settings [22], all of which are supported using HoloKinect. For this work, we will be focusing on the narrow field of view depth capture mode (90° horizontal × 59° vertical) at a resolution of 320 × 288 (binned down from 640 × 576) and the 1280 × 720 color capture mode. The Azure Kinect’s built-in microphone array captures the audio.

### 2.3. Preprocessing

HoloKinect allows two main options for data preprocessing: transforming the depth map to the perspective of the color camera, or transforming the color map to the perspective of the depth camera. This work focuses on transforming the depth map to the perspective of the color camera since it provides the higher native sensor resolution. We further elected to take the Kinect’s 640 × 576 depth map and perform 2 × 2 binning to reduce the resolution to 320 × 288. Although this reduces the effective resolution, it also lessens the apparent sensor noise. The removal of high-frequency information via binning also assists in the eventual compression of the depth data, as is further discussed in Section 4.3. After binning, the 320 × 288 depth map is then transformed to the 1280 × 720 resolution and perspective of the color camera capture. The binning and transformation is performed using the Microsoft Azure SDK, which uses the Kinect’s calibration and linear interpolation to effectively transform and upscale the depth map to the color camera’s perspective and resolution.

Since our depth encoding method performs more precisely when encoding a more limited depth range, we filter depth values that fall outside of some user-specified [$Z_{min}$, $Z_{max}$]. For example, if $Z_{min}=200$ mm and $Z_{max}=1000$ mm, all depth values less than $Z_{min}$ and greater than $Z_{max}$ will be thresholded from the preprocessed depth map. We elected to retain the full color map to increase flexibility in the postprocessing pipeline.

### 2.4. Depth Encoding

To achieve real-time 3D video communications at 30 FPS over a typical broadband connection, the 3D information acquired from the Kinect must be compressed. There have been a variety of techniques proposed for the compression of such information in 3D mesh representations [23]. For this work, however, an image-based encoding method was adopted in order to leverage existing hardware and software support for efficiently delivering compressed 2D video over the internet. Further, given that the Kinect only captures a single perspective, transmitting the entire 3D mesh may be unnecessary. Instead, the depth map is transmitted, and the resulting 3D mesh is reconstructed from the depth map on the receiving end, given that the capture device’s calibration information is known.

To encode a 1280 × 720, 16-bit depth map, *Z*, into a traditional 2D RGB image, the multiwavelength depth (MWD) encoding method [18] was utilized. This image-based depth encoding method offers (1) a relatively simple 2D encoding scheme, generating three encoded channels via pixel-wise operations on the depth map; (2) encodings that vary smoothly in intensity, making them suitable for lossy compression; and (3) simplicity from a computational perspective, making it efficient for a variety of device types. The MWD encoding method can be described mathematically as

(1) $$I_{1}\left(i,j\right)=0.5+0.5\sin\left(2\pi \times \frac{Z(i,j)}{P}\right),$$

(2) $$I_{2}\left(i,j\right)=0.5+0.5\cos\left(2\pi \times \frac{Z(i,j)}{P}\right),$$

(3) $$I_{3}\left(i,j\right)=\frac{Z\left(i,j\right)-Z_{min}}{Z_{max}-Z_{min}}.$$

In the above equations, *Z* refers to the 1280 × 720 depth map to be encoded; (i,j) refers to the location of each individual pixel; [$Z_{min}$, $Z_{max}$] refers to the user-defined depth range; and *P* refers to a user-defined *fringe width*. This fringe width ultimately determines the encoding frequency of the depth map, and it is often defined via its inverse relation to the number of periods ($n_{stairs}$) with which the user would like to encode the depth range:

(4) $$n_{stairs}=\frac{Z_{max}-Z_{min}}{P}.$$

In principle, as the number of encoding periods (i.e., number of stairs) increases, so will the encoding frequency, leading toward a higher precision encoding. There is a trade-off, however, as an increased encoding frequency causes the resultant encodings to vary in intensity more rapidly, leading toward larger file sizes and the potential for compression artifacts (this relationship will be highlighted and discussed further in Section 4.2).

**Figure 2:**
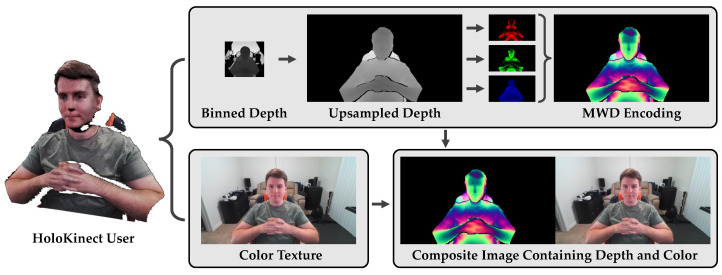
HoloKinect encoding pipeline. Depth and color maps are are collected at 30 FPS from the Microsoft Azure Kinect. In this work, we focus on a 320 × 288 depth map, binned down from a 640 × 576 depth map collected using the Kinect’s narrow field of view option. The 320 × 288 depth map is transformed and linearly interpolated to the perspective and resolution of the color camera (1280 × 720). Depth values outside of the range [$Z_{min}$, $Z_{max}$] are thresholded. Multiwavelength depth (MWD) encodes the 16-bit depth measurements into the red, green, and blue channels of a standard RGB image using Equations (1)–(3), respectively. This 1280 × 720 MWD encoding is combined side-by-side with the color texture to create a 2560 × 720 composite frame. This composite frame is then readied for video compression and transmission.

After the 1280 × 720 preprocessed depth map, *Z*, has been encoded into three single-channel encodings by Equations (1)–(3), a single three-channel RGB image can be composed. It is this RGB image, with $I_{1}$, $I_{2}$, and $I_{3}$ being placed in the red, green, and blue channels, respectively, that contains the Kinect’s encoded depth information. The depth map’s corresponding 1280 × 720 color image is then appended to the right of the MWD-encoded image to produce a 2560 × 720 composite image. The composite image—containing both depth and color information—is then ready to be compressed with a lossy video codec and transmitted to a remote participant. HoloKinect provides both CPU-based and GPU-based implementations of the MWD encoding, however, we opted to perform the encoding on the CPU to avoid the latency of data transfers to and from the GPU. Figure 2 summarizes and illustrates HoloKinect’s encoding module.

### 2.5. Video Streaming

The result of the aforementioned Acquisition, Preprocessing, and Depth Encoding modules is a continuous, 30 FPS stream of composite images that contain MWD-encoded depth and color information. Video compression was employed, enabled by the smooth nature of the MWD encoding, to facilitate the efficient two-way transmission of composite image streams between remote users over a standard internet connection. Specifically, the Agora Video SDK [20] for Unity was used to provide video compression and transmission of each user’s generated video and captured audio streams. Instead of delivering 2D webcam images, as typically occurs with a video streaming service such as Agora, we provide custom frames (the stream of composite images) for video compression and transmission. This off-the-shelf plugin serves as a low-latency WebRTC [19] solution using H.264 [24] video compression with 4:2:0 chroma subsampling. The target resolution, bitrate, and other streaming parameters are configurable with this software. It should be noted that utilizing Agora is not a specific requirement: given the modular nature of HoloKinect, custom or alternative video streaming providers could have been used instead.

### 2.6. Decoding

On a receiving client, the video stream is processed to recover the depth and color information contained within each composite frame. To prepare for the data’s eventual postprocessing and visualization, each received composite video frame is uploaded to and processed by a custom, GPU-accelerated compute shader in Unity. The right half of the composite image simply contains the transmitted color texture, and the left half contains the MWD-encoded depth map. To recover the depth map stored within this encoding, MWD decoding [18] is performed.

A high-frequency *wrapped phase* map is recovered from an MWD encoding’s red channel ($I_{1}$) and green channel ($I_{2}$) via

(5) $$\phi_{H}\left(i,j\right)=\tan^{-1}\left(\frac{I_{1}\left(i,j\right)-0.5}{I_{2}\left(i,j\right)-0.5}\right).$$

While this high-frequency wrapped phase contains the data necessary to recover the depth information, it has sharp periodic 2π discontinuities due to the arctangent function. To locate these discontinuities for correction, a reference is made to the normalized depth map stored in the MWD encoding’s blue channel ($I_{3}$). This depth map is first transformed into a low-frequency wrapped phase map by

(6) $$\phi_{L}\left(i,j\right)=I_{3}\left(i,j\right)\times 2\pi -\pi .$$

An integer *stair image*, *K*, is then computed as

(7) $$K\left(i,j\right)=\mathrm{Round}\left(\frac{\phi_{L}\left(i,j\right)\times \left(Z_{max}-Z_{min}\right)/P-\phi_{H}\left(i,j\right)}{2\pi }\right).$$

This stair image represents the integer multiples of 2π that must be added to the wrapped phase to recover the unwrapped phase via

(8) $$\Phi \left(i,j\right)=\phi_{H}\left(i,j\right)+K\left(i,j\right)\times 2\pi .$$

The decoded depth map, $Z^{\prime }$, is then recovered after scaling the unwrapped phase map:

(9) $$Z^{\prime }\left(i,j\right)=\frac{\Phi (i,j)\times P}{2\pi }.$$

Once the depth map has been recovered, it can be transformed into a 3D mesh (i.e., $X^{\prime }\left(i,j\right)$ and $Y^{\prime }\left(i,j\right)$ can be computed for each $Z^{\prime }\left(i,j\right)$) by utilizing the Kinect’s provided calibration information. More details of MWD’s procedures can be found in [18].

It should be noted that while MWD attempts to encode the depth information in a manner suitable for lossy video compression, it is not impervious to compression artifacts (especially as target bitrates decrease). Practically, video compression artifacts can result in errors within each of the decoding operations listed above. These errors occur particularly in regions that contain sharp transitions, such as along the outside edges of the captured subject. For example, if the values in the received blue channel ($I_{3}$) vary slightly due to the process of video compression, values in the resulting $\phi_{L}$ may be slightly incorrect. This may lead to incorrect values within the stair image, *K*, subsequently leading to incorrectly adjusting 2π discontinuities in the calculation of Φ. The end effect of these errors are large, visually displeasing spikes in the recovered depth map. Given the nature of the lossy video compression employed to transmit our encoded depth information, we also implement a series of postprocessing steps to prepare the reconstructed 3D mesh for visualization.

### 2.7. Postprocessing

Similar to the depth decoding, the entire postprocessing pipeline is GPU-accelerated with a Unity compute shader that manipulates a 3D mesh data structure. The first step is to sum the normalized color channels of the MWD encoding together and use a black-level threshold to remove any encoded depth pixels below a user-defined value. This ignores the encoding’s black background and prevents most minor compression artifacts from creating depth samples where none were present in the original sensor data. Additionally, any decoded depth value that falls outside of the encoding’s depth range, [$Z_{min}$, $Z_{max}$], is also discarded since these erroneous values would have been caused by compression artifacts.

Our methodology only reconstructs a single mesh, so any large depth discontinuities in neighboring vertices are visually distracting (e.g., a person in the foreground should not be connected to a wall in the background). Therefore, a depth discontinuity threshold is used to filter out any vertex that neighbors a depth discontinuity (determined by distance between neighboring vertices). This allows a single mesh to contain neighboring foreground and background objects, without unrealistically connecting them.

Oftentimes the Azure Kinect depth sensor has small holes where it failed to reliably measure the depth. This can be caused by issues such as reflective surfaces. Since this work aims to facilitate two-way 3D video conferencing, it is advantageous to attempt to fill holes caused by obstructions such as eyeglasses. Similarly, the depth sensor also may capture small surface patches that are not connected to any larger objects, especially after depth discontinuity filtering. These small patches can be removed to further improve aesthetics. We employ hole filling and small surface patch removal via a series of dilations and erosions of the depth map to create appropriate masks.

The Azure Kinect depth sensor exhibits sensor noise that often survives the encoding and decoding process. One of the best ways to improve the subjective visual appearance of this noisy depth map is to apply spatial filtering. A naive approach would be to apply a Gaussian blur to smooth the depth map. However, if iterated many times, a Gaussian blur will erode away sharper features in the depth map such as noses. Therefore, we also implemented a modified bilateral filter (similar to [25]) that takes into account depth and color similarities among neighboring vertices. Additionally, this bilateral filter is only iterated in locations where the range of depth values within the filter’s kernel does not exceed a specified threshold. This limits smoothing in regions with steep depth gradients and helps to prevent the erosion of sharper features, even when aggressive smoothing is applied.

**Figure 3:**
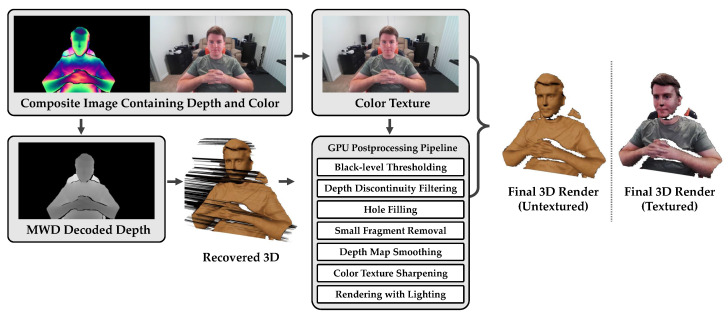
HoloKinect decoding and postprocessing pipeline. Due to the software settings we have elected to focus on in this work, Agora receives and decodes a 2560 × 720 RGB composite frame containing MWD-encoded depth and color texture information. The MWD data is decoded on a GPU-accelerated Unity compute shader using Equations (5)–(9). Decoded depth values outside of [$Z_{min}$, $Z_{max}$] are filtered out, as are pixels with brightness less than a black-level threshold. Also available within the GPU-accelerated compute shader are additional postprocessing steps, including the filtering of vertices adjacent to a depth discontinuity, depth hole filling, the removal of small depth surface fragments, and depth map smoothing. Additionally, the color texture can be sharpened using a standard sharpening kernel or using unsharp masking. The final 3D rendering is performed by the Unity game engine. This rendering can add highlights, shadows, and other visual effects derived from virtual light sources. The renderings here were generated using Unity’s High Definition Render Pipeline (HDRP) and a lit mesh shader, for example.

Although the Azure Kinect color camera can capture images at resolutions of up to 4 K, these images can often appear a bit soft. Sharpening the color map can enhance details and improve the subjective appearance of a remote participant. Both a standard sharpening kernel and unsharp masking are available to sharpen the color texture. Figure 3 illustrates HoloKinect’s decoding and postprocessing modules. In this example, the black-level threshold was 0.2, the depth discontinuity filtering threshold was 10 cm, and 5 iterations of a 3 × 3 modified bilateral depth-smoothing filter was applied.

### 2.8. Visualization

After postprocessing, the 3D mesh data structure containing the 3D coordinates and color information is handed off to Unity’s rendering engine. The rendered mesh is formed by connecting neighboring vertices into a series of triangles. Undefined depth values (discarded by any one of the postprocessing steps) are coded with not a number (NaN), which causes Unity to skip the rendering of any triangle containing an undefined depth value. The mesh’s surface normals are recomputed and updated after the final depth map postprocessing step completes. An option to use either an unlit graphics shader or a lit graphics shader is provided. The unlit shader does not interact with the game lighting in any way besides creating shadows. The apparent visual details in this mode derive mostly from the color map. Conversely, if the lit shader is chosen, the 3D mesh can be dynamically relit with new light sources to create highlights and enhance the details of the underlying geometry.

**Figure 4:**
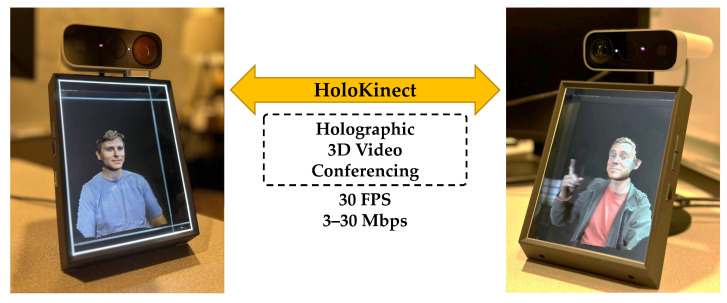
Visualization of a live two-way HoloKinect 3D video call on a pair of Looking Glass Portrait multiscopic displays. The Looking Glass Portrait provides 48 distinct views of the reconstructed 3D mesh, each with a resolution of 420 × 560. The left and right eyes see distinct views, enabling a 3D viewing effect.

The Looking Glass Unity Plugin [21] provides an adjustable capture volume for display. This volume can be adjusted in terms of its position, rotation, scale, field of view, and clipping planes. Any mesh data within this volume will be rendered into a series of 48 distinct views, each with a resolution of 420 × 560. These views are stitched together into a 3360 × 3360 quilt and transmitted over HDMI to the Looking Glass Portrait. The Looking Glass Portrait multiscopic display creates a simulated 3D holographic effect by sending out the light from these views at different angles, allowing the left and right eyes to see different views. In addition, a parallax effect can be experienced by moving horizontally across approximately 50° of valid viewing angles. While the word holographic is used in marketing materials to describe how the Looking Glass Portrait’s 48 simultaneously rendered views resemble a 3D volume to the viewer, it should be noted that this multiscopic display is distinct from displays that rely on optical diffraction and interference, such as those used in conventional holography [26,27]. Figure 4 shows a pair of Looking Glass Portrait displays in use during a live two-way HoloKinect call. The users on either end of the call are able to enjoy a glasses-free 3D video conferencing experience.

## 3. Results

### 3.1. Experimental Overview

To explore the effects of various depth and video encoding parameters on the eventual reconstruction quality of the 3D mesh, an analysis of this parameter space was performed. A reference video, containing both depth and color maps, was recorded with the Azure Kinect at 30 FPS for a total of 19.4 s (582 frames). For this analysis, $Z_{min}$ was fixed to 200 mm and $Z_{max}$ to 1000 mm, creating an 800 mm depth range appropriate for video conferencing with the Azure Kinect in its narrow field of view setting. The 640 × 576 depth maps were binned down to 320 × 288 and then transformed to the perspective of the color camera at a resolution of 1280 × 720. We found that these initial preprocessing steps reduced some of the inherent noise in the depth sensor and served as basic upscaling, without burdening the receiver with these tasks. These preprocessed depth data serve as the ground truth for the quantitative analyses and help to isolate the end-to-end losses of the depth encoding and video compression pipeline.

The preprocessed 1280 × 720 depth maps were encoded with MWD and then stored side-by-side with their respective 1280 × 720 color textures in standard RGB frames with total resolutions of 2560 × 720. We can use Agora with H.264 video encoding to send these frames, but Agora does not allow for fine control over the video compression parameters. Further, network inconsistencies would have added confounding factors to the quantitative analysis. In order to precisely and reproducibly control the video compression settings, we losslessly compressed each composite frame as a PNG image and then leveraged FFmpeg [28] to perform offline encodings with both the H.264 and H.265 video codecs. Both full 4:4:4 chroma sampling and 4:2:0 chroma subsampling of the Y’CbCr color space were performed. Through iterative estimation of the constant rate factor (CRF) needed to achieve the target average bitrates, video encodings were produced at bitrates from 3 to 30 Mbps, in 1 Mbps increments. Encodings produced through this method achieved average bitrates with errors ±1% compared to the target. It should be noted that no audio information was included in these encodings. Lastly, the $n_{stairs}$ parameter of the MWD encoding was varied from 1 to 20, in increments of 1, in order to find the optimal range in the intended use case. In all, the parameter space analyzed included 2 video codecs, 2 chroma sampling methods, 28 target bitrates, and 20 variations of MWD encoding. In total, 2240 permutations of the encoding and video compression parameters were explored, each used to create a depth-encoded, video-compressed version of the original reference video. The quantitative analysis of these data was implemented and performed within Unity’s compute shader system.

### 3.2. Qualitative Analysis

Each permutation of the original reference video was decoded offline with FFmpeg into PNG image frames. These image frames were ingested by the HoloKinect Unity project and processed by the same MWD decoding and postprocessing modules used to perform the live two-way 3D video streaming. A black-level threshold of 0.2 was selected, and a depth discontinuity filtering threshold of 10 cm was used. Depth values that fall outside of [$Z_{min}$, $Z_{max}$] = [200 mm, 1000 mm] were also removed.

**Figure 5:**
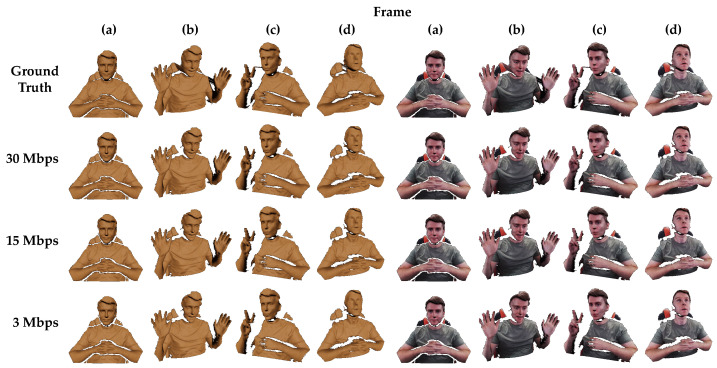
Example frames rendered with Unity’s HDRP for the ground truth data and various encoding qualities taken from several different camera views. The columns on the left half are untextured and the columns on the right are textured with color. All renderings use a lit shader and a single directional light source. The color and depth map resolutions are both 1280 × 720 in all renderings. The first row shows the ground truth data with no postprocessing. The bottom three rows show reconstruction results from videos compressed at different target bitrates with H.264 and 4:2:0 chroma subsampling. The videos contained composite frames with both color texture and MWD-encoded depth information, using $n_{stairs}$ = 6 and a depth range of 800 mm. Full videos of the reconstructed renderings shown in this figure are available as supplementary videos (Supplementary Video S1 and Supplementary Video S2).

Figure 5 shows exemplar renderings from Unity’s High Definition Render Pipeline (HDRP), both textured and untextured. Several different representative frames and camera angles have been selected. The top row shows frames from the original, uncompressed reference recording. Results from the 30, 15, and 3 Mbps video encodings follow in subsequent rows. These renderings are reconstructed from H.264 videos using 4:2:0 chroma subsampling (mirroring settings used by Agora during a live HoloKinect video call). For reasons explained in Section 4.2, $n_{stairs}$ was set to 6 in all MWD encodings for best results. Supplementary videos provide visual comparisons over the entire reference video sequence between the ground truth and reconstructions from 30 Mbps, 15 Mbps, and 3 Mbps. Supplementary Video S1 shows untextured lit renderings, Supplementary Video S2 shows textured lit renderings, and Supplementary Video S3 shows textured unlit renderings.

It can be seen that stepping artifacts present in the ground truth data (caused by 16-bit quantization) have largely been smoothed away by the video compression in all encoding qualities. Overall, despite the wide range in compression ratios, the decoded renderings all appear remarkably similar to one another. In comparison to the ground truth data in the top row, these renderings all suffer from losses around the edges of the valid depth measurement regions, likely due to ringing artifacts resulting from the sharp transitions to black in the MWD-encoded images. These errors often result in discontinuities greater than the 10 cm threshold, causing these vertices to be filtered out. The higher bitrate encodings suffer less severe ringing artifacts and thus demonstrate less edge erosion than the 3 Mbps encoding. Similar sharp transitions and subsequent vertex filtering occur in regions of steep depth changes, such as directly under the chin. Losses in this region are present in all encoding qualities and are most evident in renderings for frames (c) and (d).

Lower bitrates result in more blocking artifacts and an overall lower quality in the decoded depth maps. These differences are easiest to observe on the subject’s right hand in frame (c). Here, the lower bitrate encoding clearly suffers from more noise and an unnaturally jagged skin texture. However, these degradations in the quality of the recovered depth map would be more difficult to perceive without strong lighting effects or off-axis viewing angles. Lower bitrate videos will suffer from additional losses during frames with higher levels of motion (as can be seen in the lower bitrate reconstructions in the aforementioned supplementary videos), although these losses could largely be addressed by enabling the provided hole filling. Since the target bitrate also affects the quality of the color texture, some losses should be expected here as well. However, differences in the color texture detail and sharpness are not easily resolved in these renderings. The most obvious deterioration in the color texture quality can be seen on the shirt in reconstructions from the 3 Mbps video.

### 3.3. Quantitative Analysis of Parameter Space

**Figure 6:**
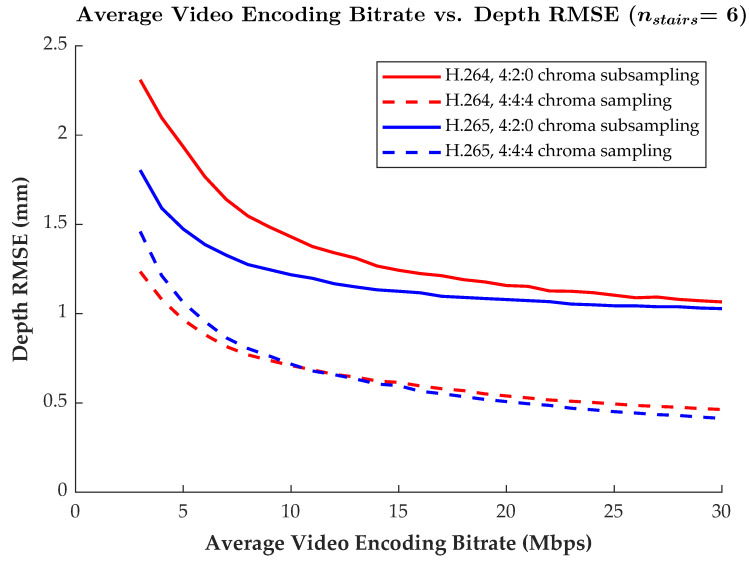
Plot of video encoding bitrate versus the RMSE in the recovered depth values. Results for the H.264 and H.265 codecs with both 4:2:0 chroma subsampling and 4:4:4 chroma sampling are included for comparison. The RMSE is measured as an aggregate over the entire 582-frame reference video encodings. The MWD encoding used $n_{stairs}$ = 6 and other settings were as described in the first paragraph of Section 3.2.

While Agora is used to provide live H.264 video compression that employs 4:2:0 chroma subsampling, HoloKinect has been designed to be modular such that alternative video streaming solutions can be integrated. To gain more insight into how different video codecs and compression parameters impact depth reconstruction quality, alternative potential streaming configurations were explored. Figure 6 shows how the root-mean-square error (RMSE) in the successfully recovered (i.e., not filtered) depth measurements changes as a function of the target average video bitrate. The RMSE metric is taken as an aggregate measure over the entire 582-frame video sequence. Results are compared between the H.264 and H.265 codecs with both 4:4:4 chroma sampling and 4:2:0 chroma subsampling.

Clearly, at any particular bitrate, the full 4:4:4 chroma sampling has substantially lower depth reconstruction RMSE when compared to the 4:2:0 chroma-subsampled results. For instance, with the H.265 codec at 15 Mbps, the 4:4:4 encoding has an RMSE of only 0.597 mm, while the 4:2:0 encoding has an RMSE of 1.125 mm, which is over 88% higher. The effect of chroma subsampling is further explored in Section 4.1.

For the 4:2:0 chroma-subsampled encodings, the more modern H.265 codec outperformed H.264 at all target bitrates. However, this difference in RMSE narrowed as the bitrate increased. Conversely, the full 4:4:4 chroma-sampled encodings showed mixed results, with the H.264 codec showing lower RMSE values for target bitrates of 10 Mbps and below, but with the H.265 codec performing slightly better at bitrates of 11 Mbps and above. Although more modern video codecs such as H.265 may outperform H.264 in most settings, H.264 remains much more ubiquitous in terms of software support and hardware acceleration.

Table 1 compares the performance of the various encoding settings across a number of quantitative measures, including RMSE, mean absolute error (MAE), Recall, and Precision. We define Recall as the number of valid decoded depth measurements recovered (after thresholding and discontinuity filtering), divided by the total number of ground truth depth measurements. We then define Precision as the number of measurements present in both the ground truth and recovered depth, divided by the total number of recovered depth measurements. The MAE values follow similar overall trends to the RMSE trend lines discussed for Figure 6, although the differences are less stark due to the nature of the MAE measure. In general, RMSE and MAE are significantly higher when using 4:2:0 chroma subsampling, but the choice between the H.264 and H.265 codecs makes less of a difference. Depth measurement Recall is significantly higher when using full 4:4:4 chroma sampling, with a 99.11% Recall using the H.264 codec with 4:4:4 chroma subsampling at 15 Mbps, but only 96.71% Recall with 4:2:0 chroma subsampling. This loss in Recall can be attributed to the losses due to the filtering of the least accurate vertices. Overall, Recall is at least 95.6% across all settings and benefits from both increases in the target average video bitrate as well as full 4:4:4 chroma sampling. Furthermore, the Precision of the depth measurement recovery was always above 99.98%. This indicates that the black-level threshold functioned as intended, preventing the creation of spurious depth measurements where none existed in the ground truth data.

## 4. Discussion

The previous sections have described the modules and quantitative performance of the HoloKinect system. This section offers further insight regarding the selection of a chroma subsampling method, $n_{stairs}$ value, and pixel binning method. Discussion of HoloKinect’s runtime performance, strengths, limitations, and future directions is also provided.

### 4.1. Chroma Subsampling

**Figure 7:**
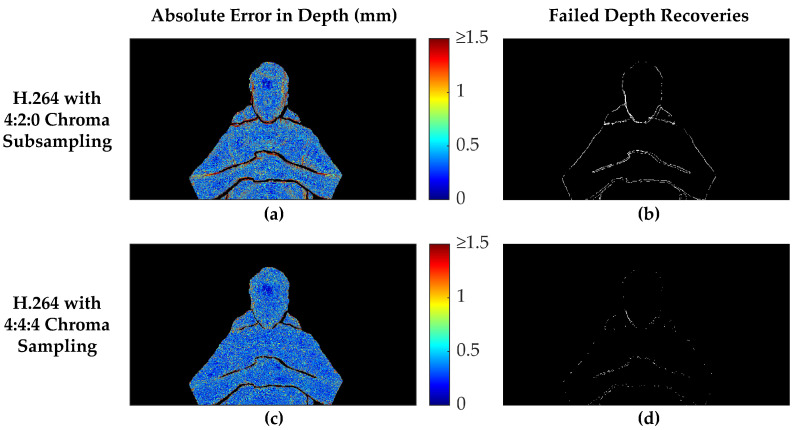
Comparison of depth reconstruction results between 4:2:0 chroma subsampling and 4:4:4 chroma sampling for an example frame. The H.264 codec was used with an average video bitrate of 15 Mbps in each case. The absolute error of the depth maps are shown in the left column, with filtered depth values shown as black. The right column shows white pixels where depth measurements that were present in the original ground truth data failed to be recovered in the final decoded and postprocessed depth map. The MWD encoding used $n_{stairs}$ = 6 and other settings were as described in the first paragraph of Section 3.2. The selected frame has a Recall of 0.9940 when using 4:4:4 chroma sampling, but only 0.9699 with 4:2:0 chroma subsampling. The recovered region in (**a**) has an RMSE of 1.030 mm and MAE of 0.5247, while (**c**) using 4:4:4 chroma sampling has a substantially lower RMSE of 0.6168 mm and MAE of 0.4123 mm.

Figure 7 presents a comparison between 15 Mbps H.264 encodings with both full 4:4:4 chroma sampling and 4:2:0 chroma subsampling. The absolute error map in the left column shows that the decoded depth map originating from the 4:2:0 encoding suffers from substantially higher errors along the edges of the valid measurement regions. In addition, areas of rapid depth changes, such as along the sides of the face and under the chin, suffer from similarly higher errors in the 4:2:0 chroma-subsampled encoding. Conversely, in the interior of the body, the absolute error maps do not differ as substantially between the two chroma sampling methods, although an overall lower error (RMSE = 0.62 mm) is apparent with the 4:4:4 encoding versus the 4:2:0 encoding (RMSE = 1.03 mm). However, the error maps and RMSE values do not include decoded depth values that were filtered out by the postprocessing steps, so these measures do not tell the full story. The right column shows where depth measurements failed to be recovered in regions that originally contained valid depth measurements in the ground truth data. Clearly, the 4:2:0 subsampled encoding suffered far more failed depth measurement recoveries, 6946 pixels for this frame versus 1384 pixels for the full 4:4:4 chroma-sampled encoding. The vast majority of these failed depth recoveries were caused by increased errors along the edges, resulting in the filtering of these values as discontinuities. However, 155 pixels along the lower-left side of the face would have been filtered as discontinuities, even if the depth measurements exactly matched the ground truth data. In this frame, the black-level thresholding did not contribute to any failed depth recoveries.

As a whole, these results agree with the way chroma subsampling operates. Since chroma subsampling was developed as a way of optimizing data compression for human perception, it is no surprise that chroma subsampling negatively affects the accuracy of pixel-precise depth measurements. Although 4:2:0 chroma subsampling of the Y’CbCr color space preserves the luma data (Y’), it explicitly discards 75% of the chroma data (CbCr), disproportionately affecting the blue and red channels when converted back into the RGB color space. Effectively, 2 × 2 pixel neighborhoods have the same CbCr values when using 4:2:0 chroma subsampling, resulting in additional error in the reconstruction of 75% of the pixels. Fortunately, these errors will not be very substantial in areas of smoothly varying depth, since neighboring pixels will have very similar depth values. However, areas of rapid depth changes suffer from increased errors.

### 4.2. Multiwavelength Depth Parameter Selection

Image-based depth encoding techniques have proved valuable as they are able to achieve large compression ratios for 3D range data by taking advantage of the more standardized fields of image and video compression. This work presents results that utilize the MWD encoding method to represent depth values within the color channels of a 2D image for lossy compression. Comparisons of MWD against previous image-based methods can be found in [29] with extensions to MWD proposed in [30,31,32]. In addition, there are alternatives to image-based depth encoding that still leverage the benefits of modern video codecs. For instance, in Project Starline [9], depth streams are stored directly within a 10-bit Y luminance channel of an H.265 video stream. Given the many methods that may exist to transport depth data, we have elected to report exact bitrates and error values so that results can be generically compared to past and future research.

Regarding the performance of MWD in this work, as mentioned in Section 2.4, there is a user-defined parameter $n_{stairs}$ that dictates the number of encoding periods used to encode depth values within some specified depth range, $\left[Z_{min},Z_{max}\right]$. Generally, for some specified range of depth values to be encoded, as the number of encoding periods increases, the fringe width *P* decreases, causing the encoding frequency to increase (these relationships can be seen in Equations (1), (2) and (4)). As the frequency increases, so will the encoding precision of depth values within the depth range [33,34]. That said, as the number of stairs (and frequency) increases, it is more difficult to compress effectively. Thus, a trade-off exists: to optimize for higher accuracy (i.e., lower error) reconstructions, the number of periods ($n_{stairs}$) can be increased, requiring a larger bitrate to retain quality; to optimize for a lower bitrate, the number of periods can be decreased at the cost of increased reconstruction error.

While the above relationships hold true in principle, there are practical considerations that arise when storing encodings produced by MWD within a 2D format for compression by a standardized lossy image or video compression algorithm. Such algorithms operate spatially, reducing redundancy *across* the image. MWD, conversely, operates by encoding *through* the depth range (in $n_{stairs}$ regions of distance *P* from $Z_{min}$ to $Z_{max}$). Therefore, as the number of stairs used to encode the depth range increases, the periods become more tightly packed within the depth range. This results in spatially closer transitions from black to white within the encoding’s $I_{1}$ and $I_{2}$ channels. Eventually, as $n_{stairs}$ increases, the transitions occur so rapidly and close together that image and video compression in these regions becomes difficult, leading to compression artifacts and large amounts of error in the recovered depth map.

**Figure 8:**
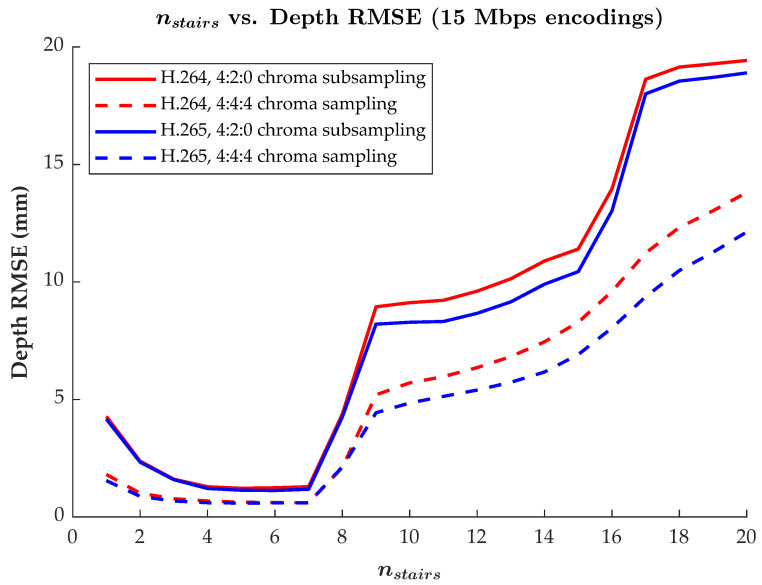
Plot of the MWD encoding parameter $n_{stairs}$ versus the RMSE in the recovered depth values. Results for the H.264 and H.265 codecs with both 4:2:0 chroma subsampling and 4:4:4 chroma sampling are included for comparison, with target average bitrates of 15 Mbps. Each RMSE is measured as an aggregate over an entire 582-frame reference video encoding. Other settings were as described in the first paragraph of Section 3.2.

The relationship of $n_{stairs}$ as it relates to reconstruction accuracy, and the practical limits of this relationship, can be seen in Figure 8. Up to when $n_{stairs}$ = 7, the trend of $n_{stairs}$ compared to reconstruction error is as expected: as $n_{stairs}$ goes up, so too does encoding precision, leading to lower rates of reconstruction error. However, after $n_{stairs}$ = 7, the amount of reconstruction error increases significantly. This is because, for our depth range of 800 mm, encoded data becomes too high of a frequency in the encoded images. Thus, these spatially high frequency regions are impacted by compression artifacts, resulting in errors in the reconstruction. In all settings, the lowest RMSE values in Figure 8 were achieved with $n_{stairs}$ between 4 and 7. For this reason, $n_{stairs}$ = 6 was consistently used throughout the previous results, as this selection offered relatively low amounts of reconstruction error with a slight margin before encoding frequencies increased too much. It should be noted that $n_{stairs}$ = 6 will not always be the best value to use as the encoding frequency is also dependent upon the depth range being encoded. For instance, a greater value of $n_{stairs}$ may more optimally encode a larger depth range by maintaining an equivalent encoding fringe width, *P* (see Equation (4)).

### 4.3. Effect of Depth Pixel Binning on Video Compression

In Section 2.2 and Section 2.3, it was mentioned that we elected to bin the 640 × 576 native resolution of the Microsoft Azure Kinect’s narrow field of view mode to a resolution of 320 × 288 before transforming to the perspective and 1280 × 720 resolution of the color camera. Although some high-frequency spatial details are lost due to binning, the sensor noise that would otherwise survive the encoding and decoding pipeline would be visually distracting and require more heavy-handed postprocessing to mitigate (which would result in similar spatial detail losses). An investigation was undertaken to quantify the effects of depth pixel binning. The experimental data consisted of two 582-frame recordings of a stationary bust that were taken in quick succession, one using 2 × 2 depth pixel binning and the other remaining unbinned. For the first recording, 2 × 2 depth pixel binning of the Microsoft Azure Kinect’s narrow field of view option results in an effective depth resolution of 320 × 288, which is transformed to the perspective and 1280 × 720 resolution of the color camera. For the second recording, the unbinned depth maps are 640 × 576 and are similarly transformed to a 1280 × 720 resolution. For the following experiments, the color texture halves of the resulting 2560 × 720 composite frames were overwritten with black pixels to eliminate the influence of the color camera data on the results. The Microsoft Azure Kinect has heuristics to determine whether or not a particular pixel’s depth measurement is valid (which can vary from frame to frame), so extra care was taken to mask out a small region of the table to the right of the bust that had unreliable depth measurements. MWD encoding was performed with $n_{stairs}$ = 6 and other settings configured as described in the first paragraph of Section 3.2. Therefore, with only the encoded depth maps of the still scene present, the depth sensor noise was the predominant source of inter-frame variations. These same recordings and preprocessing steps were used for all experiments in this subsection.

One of the motivations for electing to focus on 2 × 2 depth pixel binning results in this work was to increase the compressibility of the depth data in the selected video codecs by removing some of the sensor noise. The intended use case of HoloKinect is 3D video conferencing, which typically would be performed seated and relatively stationary. Therefore, limiting the frame-to-frame variations caused by sensor noise can have a substantial effect on the amount of compression that can be achieved at a particular quality level. Constant rate factor (CRF) is a parameter that can be used in the H.264 and H.265 codecs to target a video quality level without specifying a target bitrate. Generally, with all other factors being equal, higher levels of motion (or other inter-frame variations) will increase the bitrate required to achieve a particular CRF. Table 2 explores how the average video bitrates compared for the MWD encodings of 2 × 2-binned and unbinned recordings at a set of fixed CRF values. CRF values were selected to roughly align with the CRF values used in the 3, 15, and 30 Mbps video encodings listed in Table 1, although the resultant bitrates are much lower due to the lack of motion and color data in this experiment. In all cases, the video encodings derived from unbinned depth sensor measurements resulted in 2.05- to 2.52-times higher average bitrates. A portion of these increases in video bitrate can be attributed to the cost of compressing the finer spatial details present in the unbinned recording, but the 582-frame videos are long enough to likely be dominated by the demands of compressing the inter-frame variations in an otherwise still scene.

To investigate the effects of binning from the perspective of depth map decoding, the same pair of MWD-encoded recordings were video encoded to a target average bitrate of 3 Mbps. The 3-Mbps encoding target for this still scene roughly corresponds to the CRF values used in the 30 Mbps encodings used in Table 1, with the lower bitrates being attributable to the lack of color information and motion. Two-pass, offline video encoding and decoding was performed with FFmpeg using the H.264 and H.265 codecs on MWD encodings of both the 2 × 2-binned and unbinned recordings. The resulting frames were ingested using the HoloKinect pipeline for analysis against their respective ground truth recordings, using the same postprocessing settings mentioned in the first paragraph of Section 3.2. Results are shown in Table 3. These results show that for the same target video bitrate, binning results in lower reconstruction errors in the decoded depth data. This is due to the binned depth data effectively containing less high-frequency information within each frame (spatial) and across all the frames (temporal). The unbinned depth data was not able to be as accurately reconstructed, showing that some of its finer details were lost during the compression process. Although the recovered depth RMSE is approximately 0.2 mm larger for the unbinned depth sensor data, this difference is not large enough that it would preclude the use of the unbinned sensor data when adequate video bandwidth is available.

While we clearly show that depth pixel binning improves compressibility when using the Microsoft Azure Kinect, this configuration is not a requirement. Primarily, this decision was made to increase the compressibility and to improve the qualitative results by preserving less visual noise. In use cases that are neither bandwidth-constrained nor concerned with sensor noise, it may be desirable to use the unbinned sensor data. This setting is easily toggled within HoloKinect.

### 4.4. Runtime Performance

HoloKinect is designed to keep pace with the 30 FPS capture rate of the Microsoft Azure Kinect. However, much higher frame rates are achievable in practice. Table 4 breaks down how long major processing steps in the pipeline take when executed in the Unity editor. Overall, the total time required to create and transmit a composite video frame is approximately 9.06 ms on a high-end desktop PC. Generally, we favor performing MWD encoding on the CPU, as was done in this case, in order to avoid needing to transfer data to and from the GPU. MWD encoding on the CPU takes approximately 3.143 ms, but that same operation on the GPU requires 5.144 ms due to the data transfer overhead. The total time to MWD-decode and postprocess a composite frame using the settings listed in Table 4 is approximately 4.00 ms. GPU-accelerated operations, encompassing all steps in the postprocessing pipeline, are very fast and each take approximately 0.2 ms per dispatch. For instance, smoothing the depth map via 5 passes of a 3 × 3 modified bilateral filter takes only 0.838 ms in total. Most of the 4-ms decoding and postprocessing time is actually occupied by the 2.418-ms transfer of the composite frame to the GPU memory. Note that these processing activities are not additive, since they do not always occur during each Unity frame interval (only during Unity frames where Agora sends or receives a composite frame). Additionally, these timings are not comprehensive, since other activities such as rendering the 48 distinct viewing angles for the Looking Glass Portrait take up a large proportion of the processing time. Only the average frame time of 9.172 ms represents the overall performance, equating to an average frame rate of 109.02 FPS.

Although Table 4 represents a recommended, moderate-demand configuration for HoloKinect, it is worth discussing the system’s performance with other settings. For instance, if aggressive depth map hole filling and small patch removal is added (using a total of 110 dilation and erosion operations), this adds 8.076 ms to the postprocessing time, lowering the average frame rate to 80.28 FPS. If standard 3 × 3 Gaussian smoothing of the depth map is performed instead, 5 passes of the smoothing operation would only take 0.507 ms. A standard 3 × 3 color sharpening kernel pass takes 0.233 ms, very similar to the performance of unsharp masking. However, we could make one change that would dramatically alter the performance characteristics of the system. If the same settings used in Table 4 are simply displayed on a 1080p monitor instead of a Looking Glass Portrait, the average frame rate is 684.68 FPS. This is because rendering the 48 views of the Looking Glass Portrait adds approximately 4.897 ms to the processing time of every single frame rendered by Unity, when compared to a single 1080p rendering. When restricting frame time analysis to Unity frames that do not include any processing of RGB-D data, these frames only take 0.870 ms on a 1080p display but take 5.767 ms when outputting to the Looking Glass Portrait. There are performance optimizations available for the Looking Glass Portrait capture volume. For instance, it can be configured with view interpolation to render as few as 6 distinct views.

**Figure 9:**
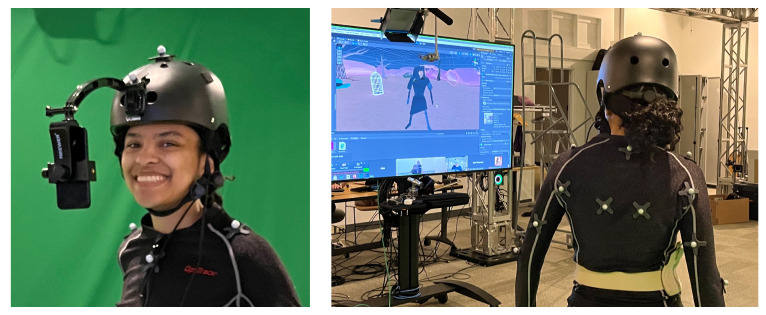
HoloKinect components used in a live theater production by the UIXR Studio at the University of Iowa. An iPhone TrueDepth sensor is used to capture RGB-D data of a performer’s face. This RGB-D data is encoded and compressed as detailed in this paper, transmitted by Agora, and finally processed by the HoloKinect pipeline on the receiving device. The reconstructed facial mesh is registered onto a digital avatar, which is controlled by the performer via an OptiTrack motion capture setup. Audience members will participate in this production remotely in Meta Quest 2 virtual reality headsets.

The core elements of HoloKinect are even able to run well on mobile devices. In Figure 9, we show an adapted version of the HoloKinect pipeline used in a live, interactive theater project. In this project, an iPhone captures facial RGB-D data, creates MWD-encoded composite frames, and transmits these data to remote audience participants via Agora. The remote participants use Meta Quest 2 virtual reality headsets to decode the data into a facial mesh, including basic postprocessing steps such as black level thresholding and depth discontinuity filtering. All of this runs seamlessly, maintaining a steady 30 FPS transmission and receiving rate, without consuming an inordinate proportion of the Quest 2’s limited computational resources.

### 4.5. Strengths and Limitations

HoloKinect presents a bandwidth-efficient, simple-to-use solution for two-way 3D video conferencing that uses affordable, off-the-shelf hardware. The integration with the Looking Glass Portrait provides an accessible and encumbrance-free method of enjoying the 3D video conferencing. The MWD encoding allows it to leverage mature, hardware-accelerated video compression codecs such as H.264. This allows for very modest bandwidth requirements, with high-quality reconstructions at 30 Mbps and very usable results at 3 Mbps. Although depth map reconstruction errors are more prevalent at lower bitrates, RMSE values of the recovered regions remain acceptably low. The software is highly configurable and open-source, so it can be adapted for many different use cases. The Unity game engine grants an advanced slew of lighting and rendering technologies, along with a very user-friendly interface. This Unity backbone also enables many components of HoloKinect to be highly portable and easily adapted for use on other supported platforms. For instance, Section 4.6 describes how HoloKinect was used to stream depth data from an iPhone to a consumer-grade VR headset.

While successful in providing an end-to-end 3D video conferencing solution, the current HoloKinect implementation is not without its limitations. For instance, it focuses on using only a single depth camera, so it only transmits a single mesh. Although this was an intentional choice for the sake of accessibility, it does limit the quality and completeness of the 3D data available for transmission. Additionally, the MWD encoding and lossy video compression inevitably degrade the depth and color data to some degree, although these losses are not always visually obvious. These data losses are exacerbated by the 4:2:0 chroma subsampling of the current Agora implementation. However, Agora makes the project more platform-agnostic than it would otherwise be, with video streaming support on Windows, macOS, iOS, and Android. HoloKinect currently processes data on a frame-by-frame basis, but temporal smoothing of the depth data would likely be helpful in reducing the impact of sensor noise and compression artifacts on the final visualization. Finally, although HoloKinect allows the final mesh to be affected by a virtual light source, this does not change the real lighting baked into the original color texture. Therefore, further work would need to be implemented to more accurately re-light the mesh. In [14], techniques were pioneered to infer the reflectance maps of a volumetric capture and dynamically relight a subject, but this required specialized capture hardware and lighting.

### 4.6. Potential Applications

The initial use case for HoloKinect is to facilitate 3D video conferencing between two individuals using a pair of Looking Glass Portrait displays. However, this system is easily adapted to other application areas. Virtual and augmented reality has traditionally resorted to simplified digital avatars, but ever-growing system capabilities make true 3D capture and transmission a tantalizing possibility. With some very simple modifications, HoloKinect can facilitate 3D telepresence in a virtual reality headset. Figure 9 shows a modified version of HoloKinect in a live theater environment where an iPhone captures 3D facial data of a performer and broadcasts this mesh to the remote audience participants.

In robotics, depth information is critical to mapping and interacting with the world. HoloKinect’s depth streaming solution provides an efficient, low-bandwidth platform to stream this information for remote control and data analysis. The Azure Kinect is already widely used in robotics applications and is fully supported in HoloKinect, but the open-source and modular nature of HoloKinect make it easy to adapt for other depth sensing solutions such as structured-light 3D cameras and lidar sensors. A hybrid RGB-D streaming solution was explored in [35], intended for use by urban search and rescue robots and drones. However, this approach used lossless depth compression methods on a frame-by-frame basis, increasing the computational burden and required bitrates. If adapted for use in search and rescue robotics, HoloKinect would be immediately useful for streaming RGB-D data, being able to leverage onboard video encoding hardware and vary the transmission bitrate to match the current wireless connection quality.

### 4.7. Future Work

Currently, HoloKinect performs only basic data preprocessing prior to transmission, with the option to perform more computationally burdensome postprocessing steps on the receiving end, if desired. However, it may be beneficial to perform additional preprocessing if the receiver is more computationally limited than the sender, or if a more powerful intermediate server is used to process the data. Depth data processing steps similar to those used in the postprocessing pipeline of HoloKinect could be implemented as a preprocessing step. This would lessen the demand on the final receiving device and allow the processing to be tailored specifically for the depth sensor noise. Additionally, since losses are inevitable in the depth and video encoding process, we may be able to lessen these losses by pre-distorting the inputs. One could envision a lightweight neural network implementation constructed to minimize end-to-end errors in the depth maps with prior knowledge of the encoding and compression methodology, similar to the work done in [36].

Additionally, as components of HoloKinect are adapted for mobile devices such as VR and AR headsets, it may be beneficial to decouple the depth and color resolutions. In the current work, the depth map and color texture resolutions were forced to be equal in order to make processing on the receiving end more practical to implement. However, if the end device is very limited and is not able to perform substantial postprocessing, then this decision may be counterproductive. Computationally limited devices will likely utilize an unlit mesh shader, and therefore derive almost all of the mesh detail from the color texture. Therefore, instead of embedding the color texture into the vertices of the mesh, it would be beneficial to allow a higher resolution color texture to be used in conjunction with a lower resolution depth map.

## 5. Conclusions

This paper has presented HoloKinect, an open-source and accessible GPU-accelerated platform for live two-way 3D video conferencing with hologram-like visualizations. HoloKinect’s depth encoding and postprocessing schemes enable the use of lossy, hardware-accelerated 2D video compression algorithms such as H.264. By leveraging the Microsoft Azure Kinect, ubiquitous 2D video compression, and the Looking Glass Portrait multiscopic display, HoloKinect achieves 3D video conferencing with commodity hardware over a standard broadband internet connection. A search of the depth and video encoding parameter space with 2240 permutations was performed to provide both qualitative and quantitative analysis. Both visual 3D reconstructions and numerical error rates were reported for a reference 3D video compressed from 3 to 30 Mbps. Overall, HoloKinect is a bandwidth-efficient, simple-to-use solution for two-way 3D video conferencing that utilizes affordable, off-the-shelf hardware. Further, given its open-source and modular design, HoloKinect can easily be adapted to provide efficient 3D video streaming to any number of applications.

## Figures and Tables

**Table 1:** Quantitative depth reconstruction results at select bitrate targets. All quantitative measures are taken as aggregates over the entire 582-frame reference video encodings. All MWD encodings used $n_{stairs}$ = 6 and other settings were as described in the first paragraph of Section 3.2.

Average Bitrate	Video Codec	Chroma Subsampling	Depth RMSE (mm)	Depth MAE (mm)	Depth Recall	Depth Precision
3 Mbps	H.264	4:2:0	2.3094	0.6249	95.60%	99.98544%
		4:4:4	1.2350	0.5187	97.51%	99.98933%
	H.265	4:2:0	1.8034	0.6095	96.12%	99.99389%
		4:4:4	1.4596	0.4967	97.72%	99.99290%
15 Mbps	H.264	4:2:0	1.2431	0.5312	96.71%	99.99958%
		4:4:4	0.6149	0.4057	99.11%	99.99945%
	H.265	4:2:0	1.1254	0.5173	96.93%	99.99967%
		4:4:4	0.5966	0.3595	99.25%	99.99951%
30 Mbps	H.264	4:2:0	1.0657	0.4995	96.94%	99.99988%
		4:4:4	0.4631	0.3491	99.54%	99.99982%
	H.265	4:2:0	1.0281	0.4865	97.05%	99.99981%
		4:4:4	0.4133	0.3051	99.56%	99.99992%

**Table 2:** Video compression results at select constant rate factors (CRF) of a still scene of a bust, using both 2 × 2-binned and unbinned depth sensor data. Two 582-frame recordings of a stationary bust were taken in quick succession, one using 2 × 2 depth pixel binning and the other remaining unbinned. The processing of these data are described in the first paragraph of Section 4.3. Constant rate factors (CRF) were selected to roughly align with the CRF values used in the 3, 15, and 30 Mbps encodings listed in Table 1. All MWD encodings used $n_{stairs}$ = 6 and other settings were as described in the first paragraph of Section 3.2.

Video Codec	Chroma Subsampling	Constant Rate Factor (CRF)	Depth Pixel Binning	Average Video Bitrate (Mbps)
H.264	4:2:0	10	2 × 2-binned	1.33
			Unbinned	2.72
		13	2 × 2-binned	0.77
			Unbinned	1.59
		21	2 × 2-binned	0.23
			Unbinned	0.45
H.265	4:2:0	7	2 × 2-binned	2.99
			Unbinned	6.24
		12	2 × 2-binned	1.25
			Unbinned	2.82
		21	2 × 2-binned	0.30
			Unbinned	0.75

**Table 3:** Quantitative depth reconstruction results of a still scene of a bust, using both 2 × 2-binned and unbinned depth sensor data. The 2 × 2-binned and unbinned depth sensor recordings are the same as those described in Table 2, with identical preprocessing. In this experiment, 2-pass video encoding was performed with FFmpeg to target an average bitrate of 3 Mbps. All quantitative measures are taken as aggregates over the entire 582-frame videos. All MWD encodings used $n_{stairs}$ = 6 and other settings were as described in the first paragraph of Section 3.2.

Average Bitrate	Video Codec	Chroma Subsampling	Depth Pixel Binning	Depth RMSE (mm)	Depth MAE (mm)	Depth Recall	Depth Precision
3 Mbps	H.264	4:2:0	2 × 2-binned	0.9060	0.6106	98.04%	99.98475%
		4:2:0	Unbinned	1.1086	0.7189	97.68%	99.98475%
	H.265	4:2:0	2 × 2-binned	0.9271	0.6134	98.03%	99.98315%
		4:2:0	Unbinned	1.1021	0.7182	97.68%	99.98233%

**Table 4:** Performance timings during a live HoloKinect video conferencing session. The general settings mirror those used in Section 3, with the Agora video stream having a total resolution of 2560 × 720. The MWD encoding used $n_{stairs}$ = 6 and other settings are as described in the first paragraph of Section 3.2. Additional postprocessing settings were enabled as detailed in the table, with black level thresholding being directly integrated into the MWD decoding function. Timings were each taken as a rolling average of 1000 samples. These measurements were made on a Windows 11 desktop PC equipped with an Intel Core i9-12900K (Intel Corp., Santa Clara, CA, USA) and an NVIDIA GeForce RTX 3090 Ti (NVIDIA Corp., Santa Clara, CA, USA).

Processing Activity	Processing Time
Depth-to-color camera transformation	3.703 ms
MWD encoding (on CPU)	3.143 ms
Frame transmission time (via Agora)	2.211 ms
Transfer received frame to GPU	2.418 ms
MWD decoding (on GPU)	0.209 ms
Depth discontinuity filtering	0.188 ms
Depth map smoothing (5 passes of 3 × 3 modified bilateral filter)	0.838 ms
Color sharpening (3 × 3 unsharp masking)	0.234 ms
Surface normal calculation	0.113 ms
Average frame time	9.172 ms

## Data Availability

HoloKinect and the reference RGB-D frames captured for this paper’s analysis will be made available at its publicly available GitHub repository (https://github.com/Holo-Reality-Lab/HoloKinect).

## Supplementary Materials

The following supporting information can be downloaded at: Supplementary Video S1: https://zenodo.org/record/7130148/files/Supplementary%20Video%201.mp4. Supplementary Video S2: https://zenodo.org/record/7130148/files/Supplementary%20Video%202.mp4. Supplementary Video S3: https://zenodo.org/record/7130148/files/Supplementary%20Video%203.mp4.

## Abbreviations

The following abbreviations are used in this manuscript:

AR	Augmented reality
FPS	Frames per second
Mbps	Megabits per second
MWD	Multiwavelength depth
RGB-D	Color and depth data
SDK	Software development kit
VR	Virtual reality
